# Rapid increase in Omicron infections in England during December 2021: REACT-1 study

**DOI:** 10.1126/science.abn8347

**Published:** 2022-02-08

**Authors:** Paul Elliott, Barbara Bodinier, Oliver Eales, Haowei Wang, David Haw, Joshua Elliott, Matthew Whitaker, Jakob Jonnerby, David Tang, Caroline E. Walters, Christina Atchison, Peter J. Diggle, Andrew J. Page, Alexander J. Trotter, Deborah Ashby, Wendy Barclay, Graham Taylor, Helen Ward, Ara Darzi, Graham S. Cooke, Marc Chadeau-Hyam, Christl A. Donnelly

**Affiliations:** ^1^ School of Public Health, Imperial College London, London, UK.; ^2^ MRC Centre for Environment and Health, School of Public Health, Imperial College London, London, UK.; ^3^ Imperial College Healthcare NHS Trust, London, UK.; ^4^ National Institute for Health Research Imperial Biomedical Research Centre, London, UK.; ^5^ Health Data Research (HDR) UK, Imperial College London, London, UK.; ^6^ UK Dementia Research Institute, Imperial College London, London, UK.; ^7^ MRC Centre for Global infectious Disease Analysis and Jameel Institute, Imperial College London, London, UK.; ^8^ Department of Infectious Disease, Imperial College London, London, UK.; ^9^ National Heart and Lung Institute, Imperial College Healthcare NHS Trust, London, UK.; ^10^ CHICAS, Lancaster Medical School, Lancaster University, Lancaster, UK.; ^11^ Quadram Institute, Norwich, UK.; ^12^ Institute of Global Health Innovation, Imperial College London, London, UK.; ^13^ Department of Statistics, University of Oxford, Oxford, UK.

## Abstract

The unprecedented rise in SARS-CoV-2 infections during December 2021 was concurrent with rapid spread of the Omicron variant in England and globally. We analyzed prevalence of SARS-CoV-2 and its dynamics in England from end November to mid-December 2021 among almost 100,000 participants from the REACT-1 study. Prevalence was high with rapid growth nationally and particularly in London during December 2021, and an increasing proportion of infections due to Omicron. We observed large falls in swab positivity among mostly vaccinated older children (12-17 years) compared with unvaccinated younger children (5-11 years), and in adults who received a third (booster) vaccine dose vs. two doses. Our results reinforce the importance of vaccination and booster campaigns, although additional measures have been needed to control the rapid growth of the Omicron variant.

Since its identification in November 2021, the Omicron variant has spread rapidly across the world, driven by its ability to cause more breakthrough infections among vaccinated individuals than other variants, likely due to genetic mutations within its viral spike protein (*1*). Furthermore, vaccine-induced protection against the Delta variant (and its sub-lineages) was already found to be waning (*2*).

The REal-time Assessment of Community Transmission-1 (REACT-1) study (*3*–*5*) has been tracking the spread of the SARS-CoV-2 virus in England approximately monthly since May 2020 as England’s first wave of infections declined. REACT-1 charted the complete replacement of Alpha by Delta from round 12 (21 May to 7 June 2021) to round 13 (24 June to 12 July 2021) (*5*). With round 16 (23 November to 14 December 2021) data we document Omicron’s early spread in England.

The SARS-CoV-2 vaccination program in England evolved quickly. In September 2021 in addition to the vaccinations offered to those 16 years of age and over, children aged 12 to 15 years were offered one dose of vaccine. By 12 December 2021, the opportunity to schedule third (booster) doses had been extended to all adults (aged 18 years and over) with heightened efforts to deliver booster doses as quickly as possible. At the same time, the rollout of the vaccination program to children aged 12 to 17 years was accelerated with second doses becoming available to 12 to 15 year-olds as well as 16 and 17 year-olds (*6*).

Here we document the early detection of Omicron in England using the community-based REACT-1 study to avoid the biases arising in case incidence data, including those due to test-seeking behavior and limited testing capacity (*4*). We compare SARS-CoV-2 swab positivity in round 16 to previous rounds.

In round 16 (23 November to 14 December 2021), 803,864 randomly selected individuals aged 5 years and over in England were invited to participate. Of these, 129,534 (16.1%) registered and 97,089 (12.1%) provided a self-administered throat and nasal swab with a valid RT-PCR test result, including 661 samples (12 positives) obtained from 15 to 17 December 2021 (see supplementary materials and fig. S1). A total of 1,192 positive swabs were detected yielding an overall weighted prevalence of 1.41% (1.33%, 1.51%), the third highest observed since the start of data collection in REACT-1 (from 1 May 2020) (table S1).

A P-spline model to all REACT-1 data revealed increasing weighted prevalence during round 16 starting around 1 December 2021 (Fig. 1A). We estimated a reproduction number R = 1.09 (1.04, 1.14) for the whole of round 16 based on an exponential model for the daily weighted prevalence, assuming a gamma-distributed generation time with mean 4.6 days and standard deviation of 3.1 days (*7*). Restricting to data from December, the estimate of R was 1.19 (1.10, 1.28) (Table 1). We also found an increase in weighted prevalence in those aged 18 to 54 years with R = 1.16 (1.10, 1.23) for the whole of round 16 and R = 1.29 (1.16, 1.42) for December only (Table 1), consistent with the P-spline model (Fig. 1B).

**Fig. 1.**
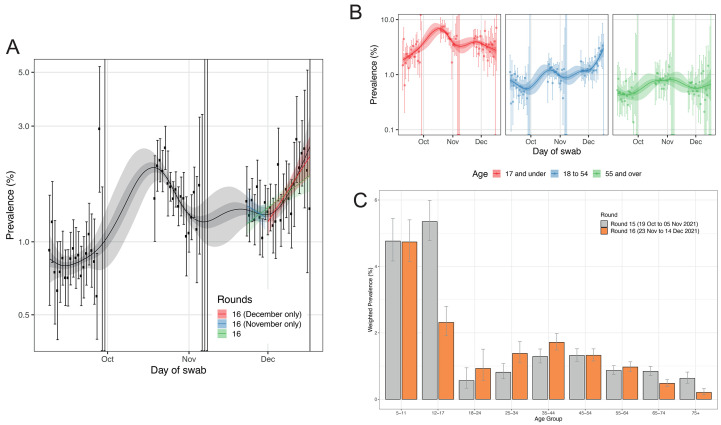
Dynamics of the prevalence of SARS-CoV-2 swab positivity in England. (**A**) Comparison of exponential model fits to round 16 overall (green), round 16 from 23-30 November (blue) and from 1 December onwards (red) in addition to a P-spline model fit to all rounds of REACT-1 (black, shown here only for rounds 14, 15 and 16). Shaded blue and red regions show the 95% posterior credible interval for the exponential models, and the shaded grey region shows 50% (dark grey) and 95% (light grey) posterior credible interval for the P-spline model. Results are presented for each day (X axis) of sampling for round 14, round 15 and round 16 and the prevalence of swab positivity is shown (Y axis) on a log scale. Weighted observations (black dots) and 95% confidence intervals (vertical lines) are also shown. (**B**) P-spline models for those aged 17 years and under (red), 18 to 54 years (blue) and 55 years and over (green). (**C**) Weighted prevalence of swab positivity by age group for round 15 and round 16. Bars show the prevalence point estimates (grey for round 15 and orange for round 16), and the vertical lines represent the 95% confidence intervals.

**Table 1.** Table of growth rates (per day), reproduction numbers and doubling/halving times (in days) from exponential model fits on data from round 16 (23 November to 14 December 2021). Data include N = 661 samples (12 positives) obtained from 15-17 December 2021. Doubling/Halving time estimates are shown only when the 95% credible intervals for R exclude 1.

		**Growth rate (per day)**	***R***	**Probability *R* > 1**	**Doubling (+) / Halving (-) time (in days)**
*Round 16*					
All positives		0.019 (0.010, 0.029)	1.09 (1.04, 1.14)	>0.99	36.1 (71.7, 24.1)
Age	Aged 17 and under	-0.019 (-0.035, -0.004)	0.91 (0.84, 0.98)	0.01	-35.7 (-19.6, *)
	Aged 18 to 54	0.034 (0.021, 0.047)	1.16 (1.10, 1.23)	>0.99	20.3 (33.6, 14.6)
	Aged 55 and over	0.005 (-0.022, 0.032)	1.02 (0.90, 1.15)	0.64	
Region	East Midlands	0.010 (-0.020, 0.040)	1.05 (0.91, 1.19)	0.75	
	West Midlands	0.019 (-0.017, 0.054)	1.09 (0.93, 1.26)	0.86	
	East of England	0.013 (-0.019, 0.043)	1.06 (0.92, 1.21)	0.79	
	London	0.060 (0.040, 0.080)	1.29 (1.19, 1.40)	>0.99	11.6 (17.5, 8.7)
	North West	0.001 (-0.032, 0.034)	1.00 (0.86, 1.16)	0.52	
	North East	-0.018 (-0.078, 0.038)	0.92 (0.68, 1.18)	0.27	
	South East	0.022 (0.000, 0.044)	1.10 (1.00, 1.21)	0.97	
	South West	-0.005 (-0.035, 0.024)	0.98 (0.85, 1.11)	0.38	
	Yorkshire and The Humber	-0.022 (-0.056, 0.012)	0.90 (0.76, 1.06)	0.10	
*Round 16 (December only)*					
All positives		0.039 (0.021, 0.057)	1.19 (1.10, 1.28)	>0.99	17.8 (32.9, 12.2)
Age	Aged 17 and under	-0.014 (-0.045, 0.017)	0.94 (0.80, 1.08)	0.19	
	Aged 18 to 54	0.058 (0.034, 0.083)	1.29 (1.16, 1.42)	>0.99	11.9 (20.2, 8.4)
	Aged 55 and over	0.011 (-0.044, 0.063)	1.05 (0.81, 1.31)	0.65	
Region	East Midlands	0.047 (-0.009, 0.101)	1.23 (0.96, 1.52)	0.95	
	West Midlands	-0.018 (-0.084, 0.045)	0.92 (0.65, 1.22)	0.29	
	East of England	0.032 (-0.028, 0.090)	1.15 (0.88, 1.46)	0.86	
	London	0.085 (0.050, 0.120)	1.43 (1.24, 1.63)	>0.99	8.2 (14.0, 5.8)
	North West	-0.004 (-0.070, 0.058)	0.98 (0.71, 1.29)	0.46	
	North East	0.067 (-0.051, 0.186)	1.34 (0.78, 2.05)	0.87	
	South East	0.047 (0.007, 0.087)	1.23 (1.03, 1.44)	0.99	14.6 (*, 7.9)
	South West	-0.027 (-0.089, 0.032)	0.88 (0.64, 1.15)	0.19	
	Yorkshire and The Humber	-0.004 (-0.072, 0.062)	0.98 (0.70, 1.30)	0.46	

*Doubling/Halving time had an estimated magnitude greater than 50 days and so represented approximately constant prevalence.

We found strong evidence of increasing weighted prevalence in London which had the highest weighted prevalence nationally at 1.84% (1.59%, 2.12%) compared with 1.23% (1.03%, 1.47%) in round 15 (Fig. 2A and table S2). For London, we estimated an R of 1.29 (1.19, 1.40) for the whole of round 16 and 1.43 (1.24, 1.63) during December only (Table 1).

**Fig. 2.**
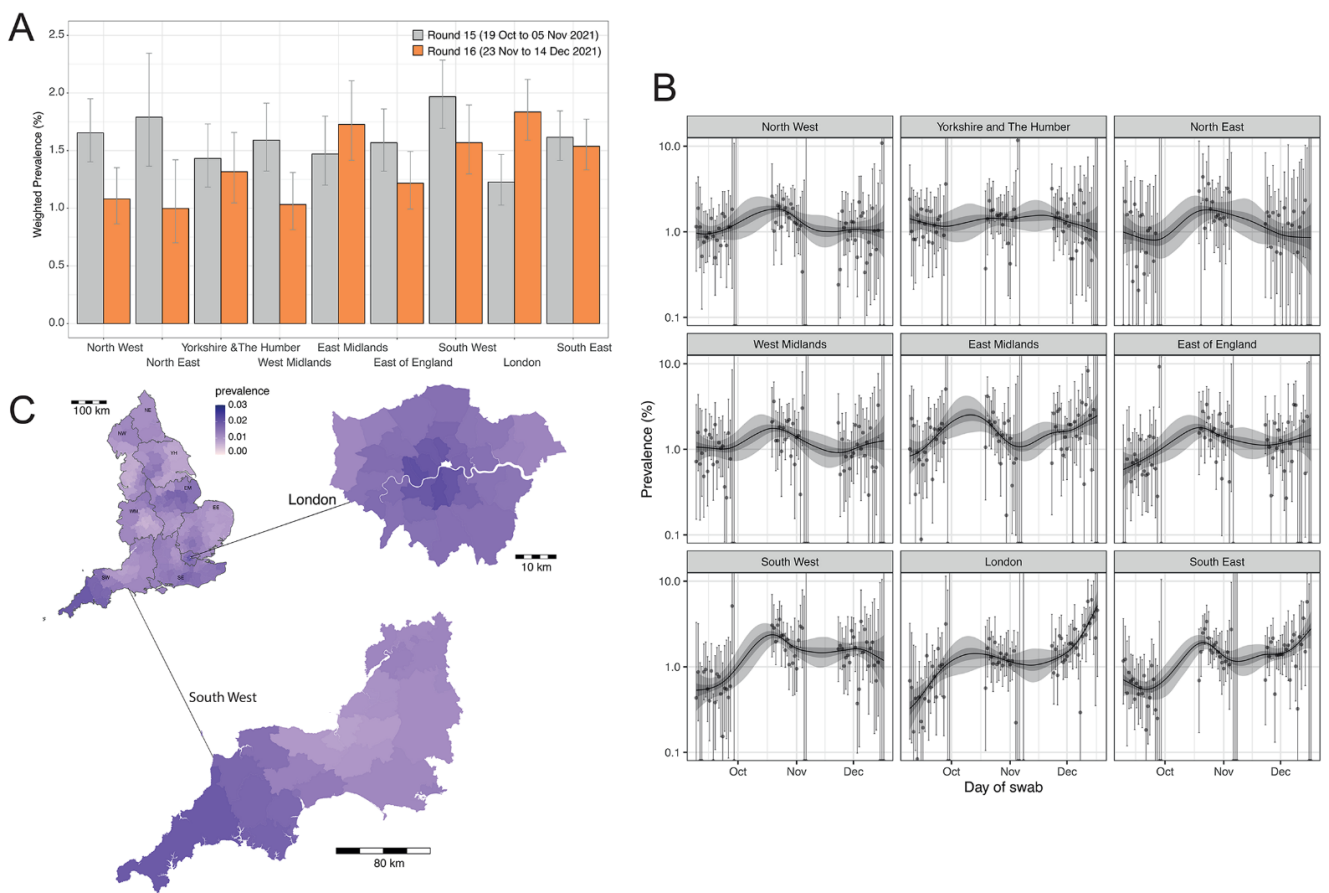
Spatio-temporal distribution of SARS-CoV-2 prevalence in England. (**A**) Weighted prevalence of swab positivity by region for round 15 and round 16 . Bars represent prevalence point estimates (grey for round 15 and orange for round 16), and the vertical lines the corresponding 95% confidence intervals. (**B**) P-spline models fit to all rounds of REACT-1 for each of the nine regions separately. Shown here only for the period of round 14, round 15 and round 16. Shaded regions show 50% (dark shade) and 95% (light shade) posterior credible interval for the P-spline models. (**C**) Neighborhood smoothed average prevalence by lower-tier local authority area for round 16. Neighborhood prevalence calculated from nearest neighbors (the median number of neighbors within 30 km in the study). Average neighborhood prevalence displayed for individual lower-tier local authorities for the whole of England and for South West and London. Regions: NE = North East, NW = North West, YH = Yorkshire and The Humber, EM = East Midlands, WM = West Midlands, EE = East of England, L = London, SE = South East, SW = South West.

Region-specific daily estimates of weighted prevalence confirmed a steep increase in London from 0.80% (0.36%, 1.75%) on 26 November to 6.06% (4.06%, 9.00%) on 14 December (Fig. 2B). A slower increase was observed in daily weighted prevalence in the South East, which reached 5.75% (2.60%, 12.22%) by 15 December 2021. At the Lower-Tier Local Authority (LTLA) level, eight of the ten highest smoothed estimates of prevalence over the whole of round 16 were in London (Lambeth, Kensington and Chelsea, Hammersmith and Fulham, Southwark, Islington, Westminster, Wandsworth, Camden) with estimates among these eight LTLAs ranging from 2.15% to 1.94% while the remaining two were in South West (Cornwall, Plymouth) with estimates of 1.94% and 1.81%, respectively (Fig. 2C).

Of the 1,192 positive swabs collected during round 16, 770 lineages were determined via viral genome sequencing with at least 50% coverage (Fig. 3, A to D, and table S3), of which 56 (7.3%) were Omicron variant and all others were Delta or Delta sub-lineages. The first swab testing positive for Omicron in REACT-1 was obtained on 3 December 2021 in London (Fig. 3B and table S4). Subsequent (N = 19) cases from 7 to 12 December 2021 were detected mainly in London and southern parts of England (Fig. 3C), and from 13 to 17 December 2021, an additional 36 Omicron infections were detected, primarily in London (Fig. 3D).

**Fig. 3.**
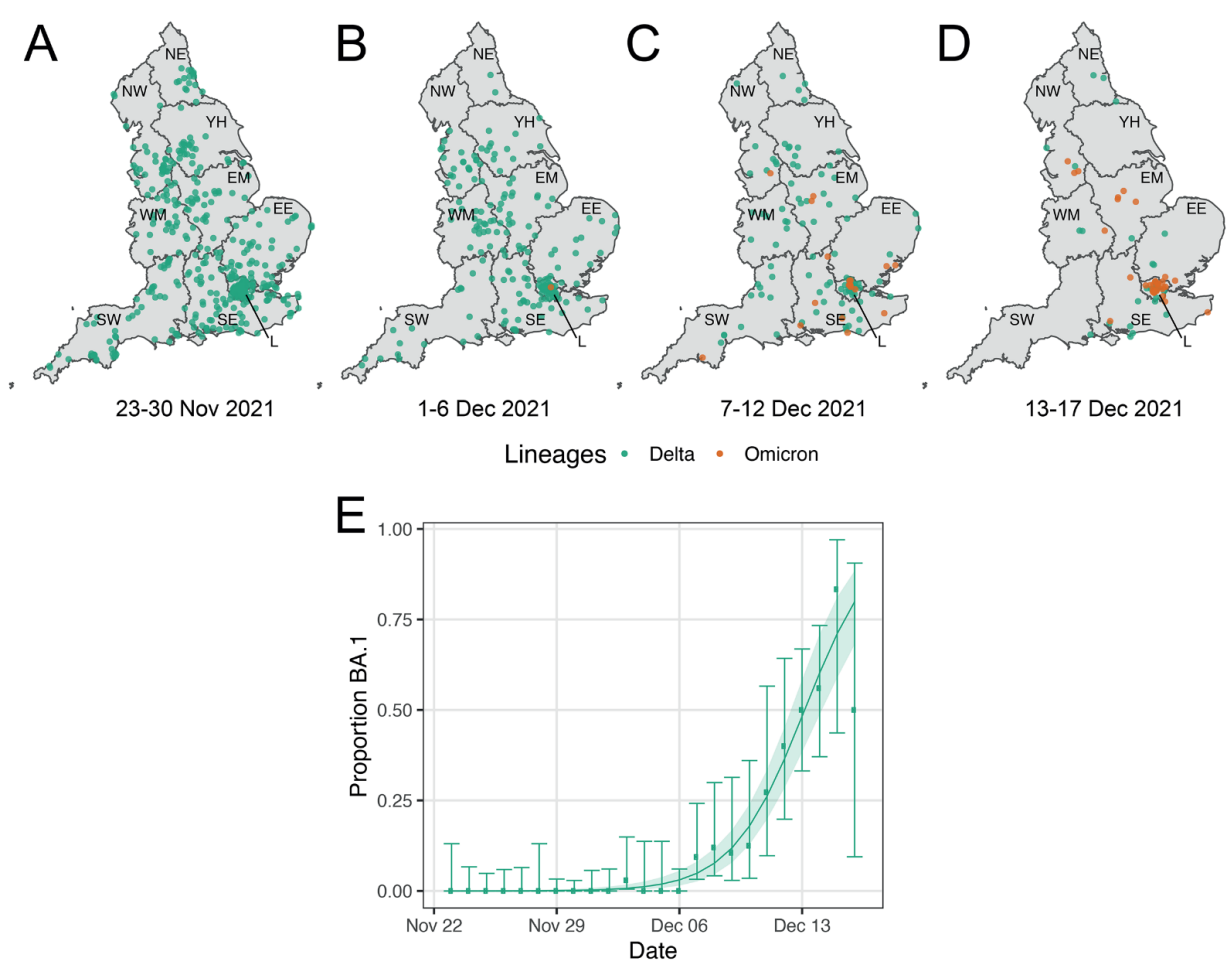
Geographical and temporal distribution of Delta and Omicron variants in England. The geographical distribution was based on the postcode of the participant’s home address (jittered to protect personal data) of the (N = 756) positive swabs with determined lineages and at least 50% genome coverage. Delta infections are presented in green, and Omicron infections in orange. Results are presented for (A) the (N=378) infections obtained from 23 November to 30 November 2021, (B) the (N=197) infections obtained in swabs from 1 December to 6 December 2021, (C) the (N=118) infections obtained in swabs from 7 December to 12 December 2021 and (D) for the (N=63) infections obtained in swabs from 13 to 17 December 2021. (E) Daily proportion of Omicron infections among positive swabs with determined lineage and at least 50% genome coverage in round 16. Point estimates are represented (dots) along with 95% confidence intervals (vertical lines). Smoothed estimates of the proportion are also shown (solid line) together with their 95% credible intervals (shaded regions). Regions: NE = North East, NW = North West, YH = Yorkshire and The Humber, EM = East Midlands, WM = West Midlands, EE = East of England, L = London, SE = South East, SW = South West.

Daily estimates of the proportion of Omicron (vs. Delta and Delta sub-lineages) rapidly increased from 6 December onwards (Fig. 3E). Smoothed estimates indicated that the proportion of Omicron infections reached over 75% by 17 December 2021. We estimate a daily increase of 61.7% (46.2%, 82.7%) in the odds of Omicron infection (vs. Delta and Delta sub-lineages), conditional on swab positivity. Assuming constant dynamics of Omicron’s (and Delta’s) transmission, we estimate 8.7 (5.4, 15.5) days for Omicron to increase from 10% to 90% of all daily infections, approximately 3.5 times faster than the estimated 31.4 (22.0, 43.9) days taken for Delta to grow from 10% to 90% against Alpha (*5*).

Based on the 56 Omicron variants detected out of 378 positives sequenced for swabs obtained from 1 to 17 December 2021, we estimate a prevalence of swab positivity for Omicron in England of 3,700 (700, 20,900) between 1 and 6 December, 142,200 (93,200, 210,000) between 7 and 12 December, and 664,800 (518,300, 803,600) between 13 and 14 December – assuming 100% sensitivity and with a weighted prevalence of 1.32%, 1.58%, and 2.14%, respectively.

Of the 56 participants with Omicron infection confirmed by sequencing, 54 (96.4%) were adults aged 18 to 54 years, for whom we found a steep increase in prevalence during round 16. The two other participants with Omicron infection were aged 65 to 74 years and none were children. Most of the participants testing Omicron-positive lived in London (N = 30, 53.7%), where there was the fastest increase in prevalence nationally during round 16. We found a difference in mean cycle threshold (Ct) values for the N-gene with mean 27.51 for Omicron compared to 25.62 for Delta-positive swabs (p = 0.015), but no difference in mean Ct values for E-gene (table S5).

The highest weighted prevalence in round 16 by age was observed among 5 to 11 year-olds who in December 2021 were not eligible for vaccination. Prevalence was 4.74% (4.15%, 5.40%) which was similar to that observed in round 15 at 4.76% (4.16%, 5.44%) (Fig. 1C and table S2). Out of the 97,089 individuals included in round 16, 84,185 (86.7%) gave consent for linkage to their vaccination data. Based on the linked data, in round 16 76.6% of participants aged 12 to 17 years had received one or two vaccine doses more than 14 days prior to swabbing (fig. S2). Between rounds 15 and 16 weighted prevalence fell in this group from 5.35% (4.78%, 5.99%) to 2.31% (1.91%, 2.80%).

Again based on the linked vaccination data, in round 16, 91.2% of participants aged 65 to 74 years had received a booster vaccine dose more than 14 days prior to swabbing as had 96.8% of those aged 75 years and over (fig. S2). Reflecting the high uptake of the booster vaccine at these ages, we observed a fall in swab positivity by 40% in those aged 65 to 74 years from 0.84% (0.72%, 0.99%) in round 15 to 0.48% (0.39%, 0.59%) in round 16, and by two-thirds in those aged 75 years and over from 0.63% (0.48%, 0.82%) to 0.21% (0.13%, 0.32%) respectively (table S2).

Weighted prevalence in round 16 was highest in larger households including 5 people at 2.73% (2.25%, 3.32%) and 6 or more people at 2.65% (2.00%, 3.50%) compared to 0.88% (0.72%, 1.09%) in single-person households; in households with one or more children at 2.43% (2.23%, 2.65%) compared to 0.85% (0.76%, 0.95%) in households without children; in those having been in contact with a confirmed COVID-19 case at 8.00% (7.25%, 8.82%) compared to 0.81% (0.73%, 0.89%) for those without such contact, and in those reporting classic COVID-19 symptoms in the month prior to swabbing at 6.96% (6.32%, 7.67%) compared to 0.62% (0.55%, 0.70%) in those without symptoms (table S6).

In England, the first Omicron infection was recorded on 27 November 2021 (*8*) [REF] and it quickly became the dominant variant, with 76% of samples processed via TaqPath laboratories having S Gene Target Failure (SGTF, an indicator of Omicron) as of 21 December 2021 (*9*). Based on these data, estimates of the regional doubling time for Omicron in England during December 2021 ranged from 1.6 to 2.5 days; London had the highest regional proportion of SGTF at 90.2%, followed by the East of England at 80.0% (*9*). At the same time, the vaccine rollout in England was accelerating both among children at ages 12 years and over and in the booster program among adults.

Against this backdrop we observed a mixed picture in round 16, characterised by i) falling prevalence of swab positivity in children aged 12 to 17 years where high levels of vaccination had been taking place, ii) high and constant prevalence in children aged 5 to 11 years who in December 2021 were ineligible for vaccination, iii) falling prevalence among older people (65 years and over) who had largely had booster vaccinations, and iv) since around 1 December 2021, rapidly rising prevalence nationally and especially in London and the South of England, coincident with the rapid rise of Omicron.

In our data 56 Omicron infections were detected up to 17 December 2021, as the Omicron epidemic became firmly established in England. This coincided with a rapidly rising proportion of Omicron compared to Delta infections, which reflected both the rapid growth of Omicron and the replacement of Delta by Omicron (*9*). Household transmission from an Omicron index case is reportedly approximately three times higher than that of Delta (*10*), which may help explain its transmission advantage. In addition, Omicron may have greater escape from immunity conferred by vaccination than Delta, with an estimated 20 to 40 times higher antibody titer required for neutralisation (*11*). Nonetheless, in vitro studies indicate that individuals receiving a booster dose of mRNA vaccine have increased neutralisation of the Omicron variant (*1*, *12*).

Our and the Coronavirus Infection Survey (CIS) data in England (*13*) suggest that Omicron infections in the first half of December 2021 predominated among young adults in which hospitalization rates are much lower than in older people, potentially biasing comparisons of variant severity despite attempts to correct for this statistically (*14*). Although Omicron appears less severe than Delta (*14*), a reduced risk of hospitalization could be rapidly offset by the observed exponential growth, which could then spill over into more vulnerable populations.

We estimate that the prevalence of swab positivity in England reached over 600,000 between 13 to 17 December. Estimates from the UK Health Security Agency indicate that there were 69,147 confirmed Omicron cases and 137,148 SGTF cases in the national testing data to 21 December 2021 (*15*). However, these figures are likely underestimates, since they depend on people presenting for testing, and unlike community-based studies such as REACT-1 and CIS, they do not include asymptomatic cases. Community-based surveillance studies can be critical in providing situational awareness and estimates of infection prevalence that are not biased by access to testing (*4*). Monitoring of hospitalizations data is also important. For example, during the period 12-21 December 2021, COVID-19 hospitalizations rose over 50% in London (*16*), a pattern not observed in areas with lower prevalence of Omicron.

Our findings in children aged 12 to 17 years amongst whom the vaccination program had been accelerated are encouraging. Since round 15 (19 October to 5 November 2021) we saw prevalence in this group fall by over a half, while it remained unchanged amongst children aged 5 to 11 years who had not been vaccinated. This strongly suggests that even a single dose of vaccine was effective against infection in children aged 12 to 17 years, although this was predominantly against Delta; indeed, we did not detect any Omicron infections through sequencing of positive swabs in children, despite them having the highest prevalence of infections overall. Our findings are similarly encouraging among older people (ages 65 years and over) among whom prevalence fell substantially, although, again, these results mostly applied to Delta infections.

Our study has limitations. Our response rate was 12.1% (i.e., returned and valid tests compared with invitations), which, despite weighted correction of our prevalence estimates (*17*), could have introduced bias into our estimates. Although all PCR-positive samples were sent for sequencing, reliable sequence data (at least 50% genome coverage) were obtained on around 60% of samples. We estimated R based on a generation time distribution estimated for Delta (*7*). To the extent that generation time may be shorter for Omicron (*7*), we may have over-estimated R during the latter part of round 16; however, our estimates of doubling time and probability that R was greater than one were unaffected. With Omicron being first observed in our data half-way through round 16, we only had two weeks of data collection in which to monitor the spread of Omicron versus Delta. The small number of sequenced Omicron cases precluded us from estimating Omicron-specific vaccine effectiveness directly.

We have documented the rapid rise of Omicron infections in England during December 2021. We found evidence for the effectiveness of vaccination in adolescents and of booster vaccinations in older adults, although this was predominantly against Delta. Thus, further data are needed to assess how well booster vaccines protect against Omicron. Moreover, given that vaccines may take up to two weeks to have their full protective effect (*18*), vaccination alone may well be insufficient to control the spread of Omicron, at least over the short term. Additional measures beyond vaccination have been needed to prevent health services in England and other countries (*19*, *20*) from being overwhelmed.

## Supplementary Materials

### This PDF file includes:

Materials and Methods Figs. S1 and S2 Tables S1 to S6 References (*22*–*30*)
