# Supplementary material for: Rapid increase in Omicron infections in England during December 2021: REACT-1 study
Source: Science. 2022 Feb 8:eabn8347. doi: 10.1126/science.abn8347 (PMC8939772; doi:10.1126/science.abn8347)
Supplement: Supplementary file 1 — Materials and Methods Figs. S1 and S2 Tables S1 to S6 References (22–30) [file science.abn8347_sm.pdf]

Science
AAAS

Supplementary Materials for

Rapid increase in Omicron infections in England during December 2021: REACT-1 study

Paul Elliott *et al.*

Corresponding authors: Paul Elliott, p.elliott@imperial.ac.uk; Christl A. Donnelly, c.donnelly@imperial.ac.uk

DOI: 10.1126/science.abn8347

This PDF file includes:

Materials and Methods
Figs. S1 and S2
Tables S1 to S6
References

Supplementary Materials: Rapid increase in Omicron infections in England during December 2021: REACT-1 study

Short title: Omicron variant in England

Materials and methods

Study population and sampling

The REACT-1 study methods are published (*3*). We have carried out cross-sectional surveys of a random sample of the population of England, aged 5 years and over, over a two- to three-week period each month since May 2020, except for December 2020 and August 2021. Here we report results for round 16 of REACT-1 (23 November to 14 December 2021, N=97,089, including 661 samples [12 positives] 15-17 December 2021) for participants with a valid self-administered throat and nose swab test result for SARS-CoV-2 by reverse transcription polymerase chain reaction (RT-PCR). (A test was recorded as positive if both N gene and E gene targets were detected or if N gene was detected with cycle threshold (Ct) value below 37.) We compare results with those obtained during round 15 (19 October to 5 November 2021, N=100,112, including 93 [all negative] from 6-8 November) and also include data from round 14 (9 to 27 September 2021, N=100,527, including 509 samples from 28-30 September) (*22*). Response rates for the three rounds (number of valid swabs returned divided by number of invitations sent out) were 12.2%, 11.7% and 12.1% for rounds 14, 15 and 16, respectively. We used the National Health Service (NHS) list of patients registered with a general practitioner in England as sampling frame, based on data held by NHS Digital who provided information on age, sex and residential postcode. Participants provided additional information on ethnicity, household size, occupation, past medical history, potential contact with a COVID-19 case, symptoms and other variables during registration and through an online or telephone questionnaire (*23*). Using the postcode of residence we linked to an area-level Index of Multiple Deprivation quintile or decile (*24*) and urban/rural status based on classification by the Office for National Statistics (*25*).

From round 1 (1 May to 1 June 2020) to round 11 (15 April to 3 May 2021) we aimed to achieve approximately equal numbers of participants in each lower-tier local authority

(LTLA) in England (N=315), but this was altered from round 12 (20 May to 7 June 2021) to invite a random sample in proportion to population size at LTLA level. This increased numbers sampled in densely populated urban areas while reducing the numbers in more rural areas. For both the original and revised sampling methods we used random iterative method (rim) weighting (*17*) correcting for age, sex, deciles of the Index of Multiple Deprivation, LTLA counts, and ethnic group, to provide estimates that were representative of the population of England as a whole, as previously described (*5*). From round 1 to round 13 (24 June to 12 July 2021) we used dry swabs which were collected from the participant's home by courier and then transported on a cold chain to a single laboratory. However, from round 14 we tested 'wet' (saline) swabs sent to the laboratory in a 1:1 randomised experiment either by courier (without cold chain) or by priority post (*22*). Since both methods provided similar results, in subsequent rounds (15 and 16) swabs were returned only using the priority postal service. However, the switch from courier to post for return of swabs meant that some samples were obtained after the nominated closing date for the study, to cover any delays in the postal service. Additional swabs received up to the cut-off date for laboratory analysis were included.

Viral genome sequencing

We sent samples that tested positive to the Quadram Institute, Norwich, UK, for viral genome sequencing using the ARTIC protocol (*26*) (version 4) for viral RNA amplification and CoronaHiT for preparation of sequencing libraries (*27*). We analysed sequencing data using the ARTIC bioinformatic pipeline (*26*) and assigned lineages using PangoLEARN (version 2021-11-4) (*28*). We searched for Omicron defining mutations down to single reads.

Data analyses

Data analyses were done using R software. We estimated weighted prevalence (and 95% credible intervals) of socio-demographic and other characteristics in round 16 and compared these to estimates from round 15.

We investigated temporal trends in swab positivity using an exponential model of growth or decay with the assumption that numbers of positives out of the total number of samples per day arose from a binomial distribution. We used day of swabbing where reported, otherwise

day of first scan of the sample by the Post Office (swabs were excluded from these analyses when neither date was available). We used a bivariate No-U-Turn Sampler with uniform prior for the probability of swab positivity on day of swabbing to estimate posterior credible intervals and the growth rate (*29*). We estimated the reproduction number R by assuming a Delta-specific gamma-distributed generation time with mean 4.6 days and standard deviation of 3.1 days REFXXX (i.e. setting the shape parameter n to 2.2 and rate parameter β to 0.48) as

$$R = \left(1 + \frac{r}{\beta}\right)^{n}.$$

To visualise trends in swab positivity over time, we fit a Bayesian penalised-spline (P-spline) model (*30*) to the daily data using a No-U-Turn Sampler in logit space. We partitioned the data into approximately 5-day sections by regularly spaced knots, and minimized edge effects by adding further knots beyond the study period. We defined fourth-order basis splines (b-splines) over the knots and guarded against overfitting by including a second-order random-walk prior distribution on the coefficients of the b-splines, with the prior penalising against changes in the growth rate unless supported by the data, as previously described (*5*). P-splines were also fit separately to three broad age groups (17 years and under, 18 to 54 years, 55 years and over) and to each region with a smoothing parameter obtained from the model fit to all the data.

We fit a Bayesian logistic regression model to the proportion of lineages identified as the Omicron variant during round 16 to obtain a daily growth rate advantage for the odds of Omicron infection compared to Delta and its sub-lineages. The daily percentage growth in the odds of omicron infection was estimated from the exponential of the daily growth rate. The time it took for the proportion of Omicron to increase from 0.1 to 0.9 was calculated from the daily growth rate, r, using the equation:

$$t_{10\%\ to\ 90\%} = \left(log\left(\frac{0.1}{1-0.1}\right) - log\left(\frac{0.9}{1-0.9}\right)\right)/r.$$

The estimated date at which Omicron reached a proportion of 0.9 was estimated from the logistic model's posterior distribution.

Comparisons were made using t-tests to test for differences between the mean N-gene Ct value and the mean E-gene Ct value for Omicron and Delta infections (where Ct>0) with *P*-values used to assess statistical significance.

We estimated geographical variation in prevalence at the LTLA level, using a neighborhood spatial smoothing method based on nearest neighbor up to 30 km. We first obtained the median number of study participants in round 16 within 30 km of each study participant, and then calculated the local prevalence for 15 members of each LTLA to estimate the smoothed neighborhood prevalence in that area.

We linked to data from the national COVID-19 vaccination programme to obtain information (with consent) on who had received one or more vaccinations and dates of vaccination. A child was considered to have been vaccinated (single dose) 14 days after administration of the vaccine and to have been unvaccinated otherwise; similarly, a child was considered to have had two doses of vaccine 14 days after the second dose. Vaccination status in adults also accounted for the same 14 days lag.

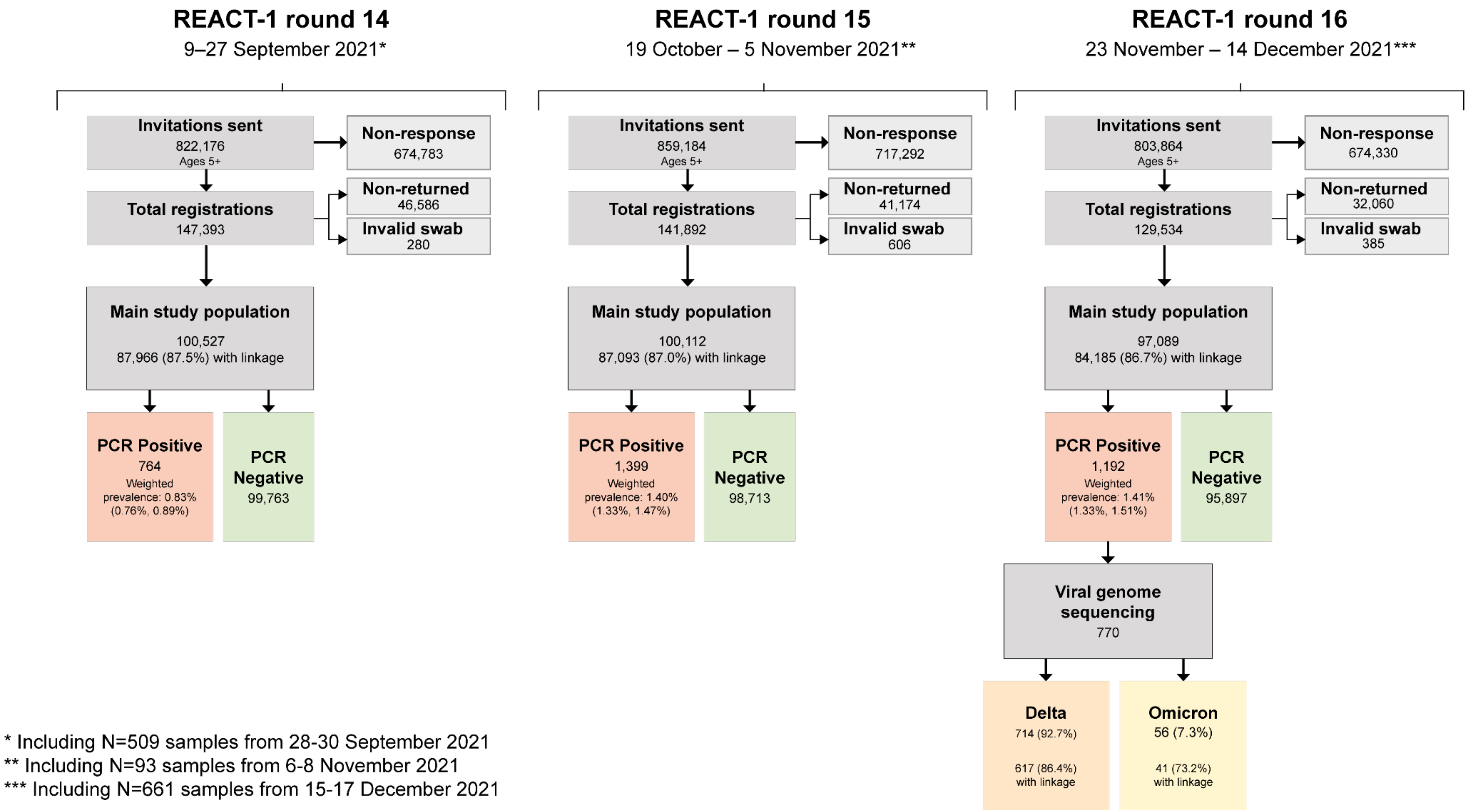

Figure S1. Flow chart showing numbers of participants in round 14 (09-27 September 2021), round 15 (19 October - 05 November 2021) and round 16 (23 November - 14 December 2021) of REACT-1.

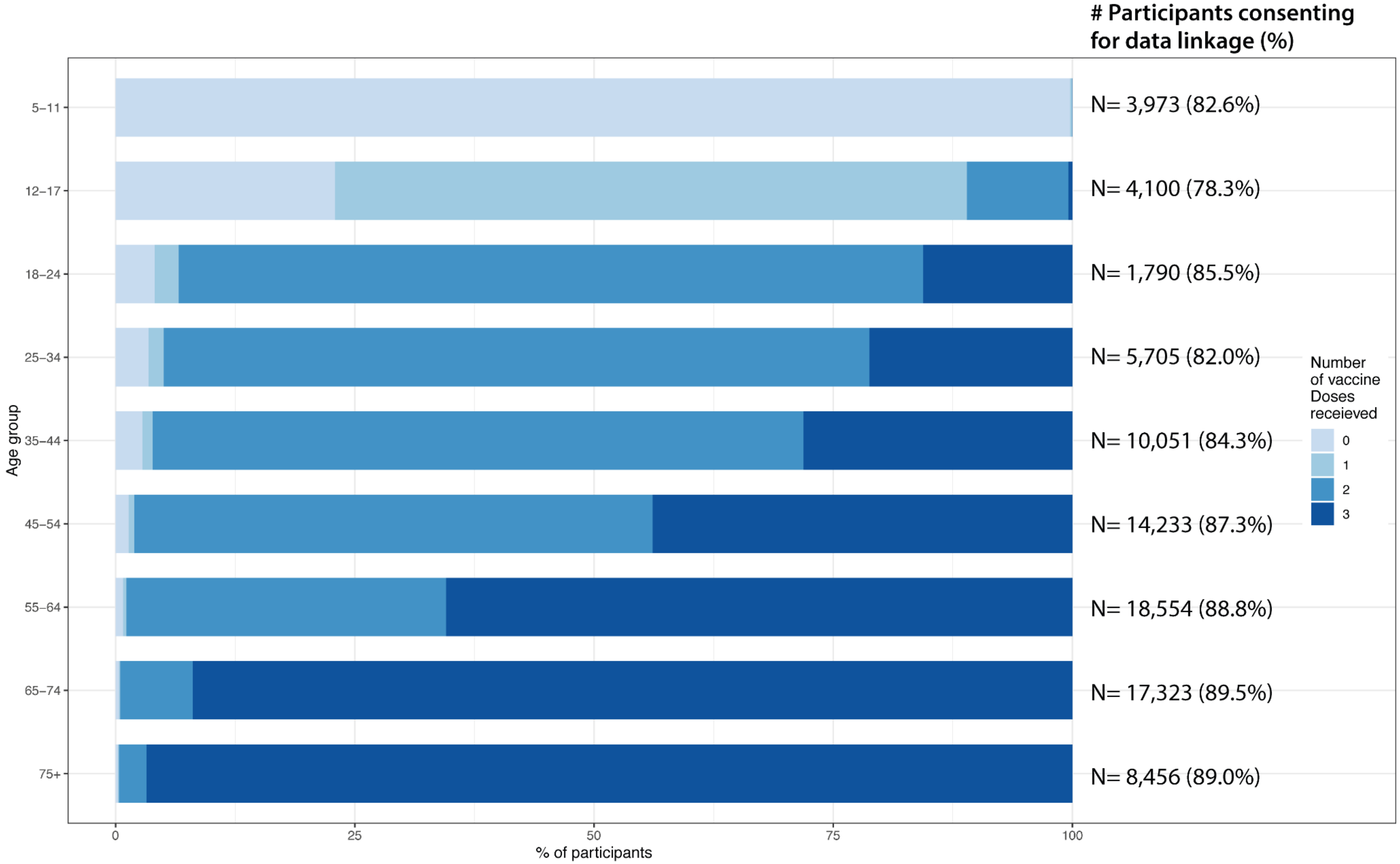

Figure S2. Proportion of unvaccinated (pale blue) participants and participants having received one, two, or three vaccine doses by age in round 16. Results are based on linked vaccination data in consenting participants. For each age group we report the total number of participants with consented linkage to vaccination data as well as (in parenthesis) the proportion it represents among all participants with valid swabs in that age group.

Table S1. Unweighted and weighted prevalence of swab-positivity from REACT-1 across rounds 1 to 16.

Round	Tested swabs	Positive swabs	Unweighted prevalence (95% CI)	Weighted prevalence (95% CI)	First sample	Last sample
1	120,620	159	0.13% (0.11%, 0.15%)	0.16% (0.13%, 0.19%)	01/05/20	01/06/20
2	159,199	123	0.08% (0.07%, 0.09%)	0.09% (0.07%, 0.11%)	19/06/20	07/07/20
3	162,821	54	0.03% (0.03%, 0.04%)	0.04% (0.03%, 0.05%)	24/07/20	11/08/20
4	154,325	137	0.09% (0.08%, 0.11%)	0.13% (0.01%, 0.15%)	20/08/20	08/09/20
5	174,949	824	0.47% (0.44%, 0.50%)	0.60% (0.55%, 0.71%)	18/09/20	05/10/20
6	160,175	1,732	1.08% (1.03%, 1.13%)	1.30% (1.21%, 1.39%)	16/10/20	02/11/20
7	168,181	1,299	0.77% (0.73%, 0.82%)	0.94% (0.87%, 1.01%)	13/11/20	03/12/20
8	167,642	2,282	1.36% (1.31%, 1.42%)	1.57% (1.49%, 1.66%)	06/01/21	22/01/21
9	165,456	689	0.42% (0.39%, 0.45%)	0.49% (0.44%, 0.55%)	04/02/21	23/02/21
10	140,844	227	0.16% (0.14%, 0.18%)	0.20% (0.17%, 0.23%)	11/03/21	30/03/21
11	127,408	115	0.09% (0.07%, 0.11%)	0.10% (0.08%, 0.13%)	15/04/21	03/05/21
12*	108,911	135	0.12% (0.10%, 0.15%)	0.15% (0.12%, 0.18%)	20/05/21	07/06/21
13	98,233	527	0.54% (0.49%, 0.58%)	0.63% (0.57%, 0.69%)	24/06/21	12/07/21
14**	100,527	764	0.76% (0.71%, 0.82%)	0.83% (0.76%, 0.89%)	09/09/21	27/09/21
15***	100,112	1,399	1.40% (1.33%, 1.47%)	1.57% (1.48%, 1.66%)	19/10/21	05/11/21
16****	97,089	1,192	1.23% (1.16%, 1.30%)	1.41% (1.33%, 1.51%)	23/11/21	14/12/21

* Sampling strategy changed for round 12 and subsequent rounds. Therefore unweighted prevalence is not directly comparable with previous rounds

** Including N=509 samples from 28-30 September. Sample handling changed in round 14. Therefore prevalence is not directly comparable with previous rounds

*** Including N=93 samples (all negatives) from 6-8 November

**** Including N=661 samples (including 12 positives) from 15-17 December

Table S2. Weighted prevalence of SARS-CoV-2 swab-positivity in round 15 and round 16 by sex, age, region, urban vs rural area, employment type, and ethnic group.

		Round 15			Round 16		
Variable		Positive	Total	Weighted Prevalence	Positive	Total	Weighted Prevalence
Sex	Male	679	44,887	1.66% (1.53%, 1.80%)	564	43,938	1.49% (1.36%, 1.64%)
	Female	720	55,223	1.48% (1.36%, 1.60%)	628	53,147	1.34% (1.23%, 1.46%)
	Unknown	0	2		0	4	
Age	05-11	222	4,693	4.76% (4.16%, 5.44%)	236	4,811	4.74% (4.15%, 5.40%)
	12-17	338	6,403	5.35% (4.78%, 5.99%)	119	5,235	2.31% (1.91%, 2.80%)
	18-24	16	3,088	0.56% (0.33%, 0.95%)	20	2,093	0.93% (0.57%, 1.51%)
	25-34	60	7,781	0.81% (0.62%, 1.07%)	89	6,961	1.38% (1.10%, 1.74%)
	35-44	160	11,933	1.29% (1.09%, 1.51%)	205	11,925	1.71% (1.48%, 1.98%)
	45-54	209	16,459	1.32% (1.14%, 1.52%)	219	16,302	1.32% (1.15%, 1.52%)
	55-64	177	20,924	0.87% (0.74%, 1.01%)	188	20,905	0.97% (0.84%, 1.13%)
	65-74	157	19,687	0.84% (0.72%, 0.99%)	94	19,353	0.48% (0.39%, 0.59%)
	75+	60	9,144	0.63% (0.48%, 0.82%)	22	9,504	0.21% (0.13%, 0.32%)
Region	South East	261	17,637	1.62% (1.41%, 1.84%)	228	16,908	1.54% (1.33%, 1.77%)
	North East	64	4,479	1.79% (1.36%, 2.34%)	43	4,289	1.00% (0.70%, 1.42%)
	North West	179	12,079	1.65% (1.40%, 1.95%)	107	11,352	1.08% (0.86%, 1.35%)
	Yorkshire and The Humber	136	9,720	1.43% (1.18%, 1.73%)	96	9,261	1.32% (1.05%, 1.66%)
	East Midlands	110	8,647	1.47% (1.20%, 1.80%)	125	8,584	1.73% (1.42%, 2.11%)
	West Midlands	135	9,943	1.59% (1.32%, 1.91%)	89	9,715	1.03% (0.81%, 1.31%)
	East of England	158	11,560	1.57% (1.32%, 1.86%)	121	11,437	1.22% (0.99%, 1.49%)
	London	160	14,847	1.23% (1.03%, 1.47%)	239	14,819	1.84% (1.59%, 2.12%)
	South West	196	11,200	1.97% (1.69%, 2.29%)	144	10,724	1.57% (1.30%, 1.90%)
Living in urban area	Yes	1,091	78,469	1.54% (1.44%, 1.65%)	956	75,982	1.43% (1.33%, 1.54%)
	No	306	21,477	1.69% (1.50%, 1.90%)	233	20,951	1.35% (1.17%, 1.55%)
	Unknown	2	166	0.79% (0.19%, 3.23%)	3	156	2.25% (0.70%, 6.98%)
Employment type	Health care or care home worker	92	7,649	1.53% (1.23%, 1.91%)	92	7,694	1.41% (1.13%, 1.76%)
	Other essential/key worker	237	14,065	1.87% (1.63%, 2.15%)	247	13,427	1.96% (1.70%, 2.25%)
	Other worker	476	38,433	1.38% (1.25%, 1.53%)	525	38,338	1.52% (1.38%, 1.68%)
	Not full-time, part-time, or self-employed	518	37,892	1.53% (1.39%, 1.68%)	303	35,679	1.06% (0.92%, 1.21%)
	Unknown	76	2,073	3.50% (2.76%, 4.44%)	25	1,951	1.19% (0.77%, 1.83%)
Ethnic group	White	1,202	87,741	1.53% (1.44%, 1.63%)	1,003	85,420	1.35% (1.26%, 1.45%)
	Asian	91	5,243	2.00% (1.58%, 2.53%)	66	4,874	1.64% (1.24%, 2.17%)
	Black	34	1,919	1.66% (1.16%, 2.37%)	38	1,833	2.10% (1.48%, 2.98%)
	Mixed	38	1,781	2.24% (1.59%, 3.14%)	31	1,600	2.03% (1.40%, 2.94%)
	Other	12	1,074	1.22% (0.67%, 2.22%)	16	984	1.81% (1.08%, 3.01%)
	Unknown	22	2,354	1.09% (0.69%, 1.71%)	38	2,378	1.96% (1.40%, 2.74%)

Table S3. Numbers and proportions of Delta (B.1.617.2), each Delta sub-lineage and Omicron (BA.1) detected in 770 positive samples with at least 50% genome coverage from round 16. Results are based on 1,192 positive samples.

Lineage	N (770)	Proportion
AY.111	5	0.006 (0.003 , 0.015)
AY.116	5	0.006 (0.003 , 0.015)
AY.120	8	0.010 (0.005 , 0.020)
AY.121	1	0.001 (0.000 , 0.007)
AY.122	9	0.012 (0.006 , 0.022)
AY.122.1	5	0.006 (0.003 , 0.015)
AY.125	2	0.003 (0.001 , 0.009)
AY.126	1	0.001 (0.000 , 0.007)
AY.127	1	0.001 (0.000 , 0.007)
AY.21	1	0.001 (0.000 , 0.007)
AY.25	2	0.003 (0.001 , 0.009)
AY.29.1	1	0.001 (0.000 , 0.007)
AY.33	1	0.001 (0.000 , 0.007)
AY.34	5	0.006 (0.003 , 0.015)
AY.34.1	1	0.001 (0.000 , 0.007)
AY.36	4	0.005 (0.002 , 0.013)
AY.39	1	0.001 (0.000 , 0.007)
AY.4	299	0.388 (0.355 , 0.423)
AY.4.2	95	0.123 (0.102 , 0.148)
AY.4.2.1	24	0.031 (0.021 , 0.046)
AY.4.2.2	11	0.014 (0.008 , 0.025)
AY.4.2.3	3	0.004 (0.001 , 0.011)
AY.42	4	0.005 (0.002 , 0.013)
AY.43	99	0.129 (0.107 , 0.154)
AY.44	1	0.001 (0.000 , 0.007)
AY.46	6	0.008 (0.004 , 0.017)
AY.46.5	6	0.008 (0.004 , 0.017)
AY.5	23	0.030 (0.020 , 0.044)
AY.5.3	3	0.004 (0.001 , 0.011)
AY.59	1	0.001 (0.000 , 0.007)
AY.6	7	0.009 (0.004 , 0.019)
AY.75	1	0.001 (0.000 , 0.007)
AY.87	1	0.001 (0.000 , 0.007)
AY.9	2	0.003 (0.001 , 0.009)
AY.9.1	3	0.004 (0.001 , 0.011)
AY.9.2	1	0.001 (0.000 , 0.007)
AY.90	1	0.001 (0.000 , 0.007)
AY.98	31	0.040 (0.029 , 0.057)
AY.98.1	2	0.003 (0.001 , 0.009)
B.1.617.2	37	0.048 (0.035 , 0.066)
BA.1	56	0.073 (0.056 , 0.093)

Table S4. Numbers of RT-PCR tests, positive tests, and Delta and Omicron infections identified by sequencing with 50% genome coverage (all Delta or Omicron) by day of swabbing.

Day of swab	Total tests	Positive tests	Positive tests with at least 50% coverage	Delta	Omicron*
2021-11-23	2,579	35	18	18	0
2021-11-24	5,293	62	38	38	0
2021-11-25	6,821	85	53	53	0
2021-11-26	6,013	68	43	43	0
2021-11-27	3,549	56	39	39	0
2021-11-28	2,448	26	18	18	0
2021-11-29	12,537	114	79	79	0
2021-11-30	10,798	126	90	90	0
2021-12-01	6,428	79	45	45	0
2021-12-02	5,868	70	42	42	0
2021-12-03	4,964	56	34	33	1
2021-12-04	2,488	27	17	17	0
2021-12-05	1,383	25	17	17	0
2021-12-06	5,279	55	42	42	0
2021-12-07	3,846	55	32	29	3
2021-12-08	3,227	43	25	22	3
2021-12-09	2,367	31	19	17	2
2021-12-10	2,776	27	16	14	2
2021-12-11	1,281	18	11	8	3
2021-12-12	798	17	15	9	6
2021-12-13	2,975	50	30	15	15
2021-12-14	1,556	33	25	11	14
2021-12-15	442	8	6	1	5
2021-12-16	163	3	2	1	1
2021-12-17	56	1	0	0	0
Unknown	1,154	22	14	13	1
Total	97,089	1,192	770	714	56

* Two additional Omicron cases were detected based on their mutation but did not pass quality control (with genome coverage<50%)

Table S5. Mean cycle threshold (Ct) values (standard deviation) for the N-gene and E-gene for Delta and Omicron positive swabs (where Ct>0) and *P*-value

	N-gene			E-gene		
Lineage	Number with N-gene detected and Ct>0	Ct value mean (sd)	*P*-value	Number with E-gene detected and Ct>0	Ct value mean (sd)	*P*-value
Delta	707	25.62 (4.78)		706	24.15 (4.93)	
Omicron	56	27.51 (5.49)	0.015	56	24.78 (5.29)	0.390

Table S6. Weighted prevalence of SARS-CoV-2 swab-positivity in round 15 and round 16 by household size, number of children in the household, contact with a COVID-19 case, symptom status and neighborhood deprivation.

		Round 15			Round 16		
Variable		Positive	Total	Weighted Prevalence	Positive	Total	Weighted Prevalence
Household size	1	132	16,997	0.78% (0.65%, 0.94%)	122	16,657	0.88% (0.72%, 1.09%)
	2	318	39,624	0.83% (0.74%, 0.94%)	287	39,443	0.77% (0.68%, 0.88%)
	3	283	17,579	1.76% (1.54%, 2.00%)	226	16,626	1.40% (1.22%, 1.62%)
	4	421	17,774	2.48% (2.23%, 2.74%)	362	17,026	2.29% (2.04%, 2.57%)
	5	178	5,752	3.12% (2.66%, 3.66%)	137	5,206	2.73% (2.25%, 3.32%)
	6+	67	2,386	2.95% (2.27%, 3.82%)	58	2,131	2.65% (2.00%, 3.50%)
Number of children in the household	0	491	66,590	0.74% (0.67%, 0.81%)	486	65,184	0.85% (0.76%, 0.95%)
	1+	644	27,239	2.62% (2.41%, 2.85%)	621	26,709	2.43% (2.23%, 2.65%)
	Unknown	264	6,283	4.22% (3.72%, 4.78%)	85	5,196	1.69% (1.35%, 2.12%)
COVID case contact	No	595	80,225	0.83% (0.75%, 0.90%)	529	75,990	0.81% (0.73%, 0.89%)
	Yes, contact with a confirmed/tested COVID-19 case	554	6,509	9.13% (8.35%, 9.96%)	463	6,048	8.00% (7.25%, 8.82%)
	Yes, contact with a suspected COVID-19 case	67	1,448	5.04% (3.88%, 6.51%)	51	1,523	3.29% (2.40%, 4.51%)
	Unknown	183	11,930	1.63% (1.39%, 1.91%)	149	13,528	1.25% (1.04%, 1.49%)
Symptom status	Classic COVID symptoms*	655	8,351	7.84% (7.22%, 8.51%)	525	7,818	6.96% (6.32%, 7.67%)
	Other symptoms	208	15,634	1.47% (1.27%, 1.70%)	211	15,377	1.52% (1.31%, 1.76%)
	No symptoms	357	64,231	0.67% (0.60%, 0.75%)	308	60,413	0.62% (0.55%, 0.70%)
	Unknown	179	11,896	1.60% (1.36%, 1.88%)	148	13,481	1.24% (1.03%, 1.49%)
Deprivation	1 Most deprived	174	11,556	1.70% (1.45%, 1.99%)	138	11,127	1.47% (1.21%, 1.78%)
	2	215	16,850	1.41% (1.22%, 1.63%)	245	16,263	1.61% (1.40%, 1.84%)
	3	283	21,096	1.51% (1.33%, 1.72%)	252	20,455	1.39% (1.21%, 1.58%)
	4	327	24,130	1.55% (1.38%, 1.74%)	255	23,307	1.26% (1.10%, 1.44%)
	5 Least deprived	400	26,480	1.67% (1.51%, 1.86%)	302	25,937	1.37% (1.21%, 1.54%)

* Classic COVID symptoms: loss or change of sense of smell or taste, fever, new persistent cough

References and Notes

1. F. Schmidt *et al.*, Plasma neutralization properties of the SARS-CoV-2 Omicron variant. bioRxiv 21267646 (2021), doi:10.1101/2021.12.12.21267646

2. E. G. Levin, Y. Lustig, C. Cohen, R. Fluss, V. Indenbaum, S. Amit, R. Doolman, K. Asraf, E. Mendelson, A. Ziv, C. Rubin, L. Freedman, Y. Kreiss, G. Regev-Yochay, Waning Immune Humoral Response to BNT162b2 Covid-19 Vaccine over 6 Months. *N. Engl. J. Med.* **385**, e84 (2021). doi:10.1056/NEJMoa2114583

3. S. Riley, C. Atchison, D. Ashby, C. A. Donnelly, W. Barclay, G. S. Cooke, H. Ward, A. Darzi, P. Elliott, REACT Study Group, REal-time Assessment of Community Transmission (REACT) of SARS-CoV-2 virus: Study protocol. *Wellcome Open Res.* **5**, 200 (2020). doi:10.12688/wellcomeopenres.16228.1

4. S. Riley, K. E. C. Ainslie, O. Eales, C. E. Walters, H. Wang, C. Atchison, C. Fronterre, P. J. Diggle, D. Ashby, C. A. Donnelly, G. Cooke, W. Barclay, H. Ward, A. Darzi, P. Elliott, Resurgence of SAR S-CoV-2: Detection by community viral surveillance. *Science* **372**, 990–995 (2021). doi:10.1126/science.abf0874

5. P. Elliott, D. Haw, H. Wang, O. Eales, C. E. Walters, K. E. C. Ainslie, C. Atchison, C. Fronterre, P. J. Diggle, A. J. Page, A. J. Trotter, S. J. Prosolek, D. Ashby, C. A. Donnelly, W. Barclay, G. Taylor, G. Cooke, H. Ward, A. Darzi, S. Riley, COVID-19 Genomics UK (COG-UK) Consortium, Exponential growth, high prevalence of SARS-CoV-2, and vaccine effectiveness associated with the Delta variant. *Science* **374**, eabl9551 (2021). doi:10.1126/science.abl9551

6. “NHS opens bookings for 12-15s to get second COVID jab” (NHS England, 2021); www.england.nhs.uk/2021/12/nhs-opens-bookings-for-12-15s-to-get-second-covid-jab/.

7. S. Abbott, K. Sherratt, M. Gerstung, S. Funk, Estimation of the test to test distribution as a proxy for generation interval distribution for the Omicron variant in England. bioRxiv 22268920 (2022), doi:10.1101/2022.01.08.22268920.

8. Department of Health and Social Care, “First UK cases of Omicron variant identified” (2021); www.gov.uk/government/news/first-uk-cases-of-omicron-variant-identified.

9. UK Health Security Agency, “Omicron daily overview: 21 December 2021” (UK Health Security Agency); https://assets.publishing.service.gov.uk/government/uploads/system/uploads/attachment_data/file/1043098/20211221_OS_Daily_Omicron_Overview.pdf.

10. “SARS-CoV-2 variants of concern and variants under investigation in England: Technical Briefing 32” (UK Health Security Agency); https://assets.publishing.service.gov.uk/government/uploads/system/uploads/attachment_data/file/1042688/RA_Technical_Briefing_32_DRAFT_17_December_2021_2021_12_17.pdf.

11. "SARS-CoV-2 variants of concern and variants under investigation in England: Technical Briefing 31" (UK Health Security Agency); https://assets.publishing.service.gov.uk/government/uploads/system/uploads/attachment_data/file/1042367/technical_briefing-31-10-december-2021.pdf.

12. N. A. Doria-Rose *et al.*, Booster of mRNA-1273 vaccine reduces SARS-CoV-2 Omicron escape from neutralizing antibodies. bioRxiv 21267805 (2021), doi:10.1101/2021.12.15.21267805.

13. "Coronavirus (COVID-19) Infection Survey, early analysis of characteristics associated with the Omicron variant among Covid-19 infections, UK: 21 December 2021"; www.ons.gov.uk/news/statementsandletters/coronaviruscovid19infectionsurveyearlyanalysisofcharacteristicsassociatedwiththeomicronvariantamongcovid19infectionsuk20december2021.

14. N. Ferguson, "Report 50: Effectiveness of SARS-CoV-2 vaccines in England in 2021: a whole population survival analysis" (Imperial College London, 2021), doi:10.25561/93035.

15. "Omicron daily overview: 22 December 2021" (UK Health Security Agency); https://assets.publishing.service.gov.uk/government/uploads/system/uploads/attachment_data/file/1043466/20211222_OS_Daily_Omicron_Overview.pdf.

16. UK Coronavirus Data Dashboard (London); https://coronavirus.data.gov.uk/details/healthcare?areaType=nhsRegion&areaName=London.

17. T. Sharot, "Weighting survey results" (1986); www.redresearch.com/wp/wp-content/uploads/2016/01/Weighting-Survey-Results.pdf.

18. N. Andrews *et al.*, Effectiveness of BNT162b2 (Comirnaty, Pfizer-BioNTech) COVID-19 booster vaccine against covid-19 related symptoms in England: test negative case-control study. bioRxiv 21266341 (2021), doi:10.1101/2021.11.15.21266341.

19. Prime Minister's Office, "Prime Minister confirms move to Plan B in England" (2021); www.gov.uk/government/news/prime-minister-confirms-move-to-plan-b-in-england.

20. M. van Algemene Zaken, "Slowing the spread of the Omicron variant: lockdown in the Netherlands" (2021); www.government.nl/latest/news/2021/12/18/slowing-the-spread-of-the-omicron-variant-lockdown-in-the-netherlands.

21. doi:10.5281/zenodo.5574472

22. "Publish with data from GISAID"; www.gisaid.org/help/publish-with-data-from-gisaid/.

23. M. Chadeau-Hyam, H. Wang, O. Eales, D. Haw, B. Bodinier, M. Whitaker, C. E. Walters, K. E. C. Ainslie, C. Atchison, C. Fronterre, P. J. Diggle, A. J. Page, A. J. Trotter, D. Ashby, W. Barclay, G. Taylor, G. Cooke, H. Ward, A. Darzi, S. Riley, C. A. Donnelly, P. Elliott, SARS-CoV-2 infection and vaccine effectiveness in England (REACT-1): a series of cross-sectional random community surveys. *Lancet Respir. Med.* 10.1016/S2213-2600(21)00542-7 (2022). doi:10.1016/S2213-2600(21)00542-7

24. REACT 1 study materials; www.imperial.ac.uk/medicine/research-and-impact/groups/react-study/react-1-study-materials/.

25. "Mapping income deprivation at a local authority level" (Office for National Statistics, 2019); www.ons.gov.uk/releases/mappingincomedeprivationatalocalauthoritylevel2019.

26. "2011 rural/urban classification"; www.ons.gov.uk/methodology/geography/geographicalproducts/ruralurbanclassifications/2011ruralurbanclassification.

27. Ncov2019-artic-nf: A Nextflow pipeline for running the ARTIC network's fieldbioinformatics tools (https://github.com/artic-network/fieldbioinformatics), with a focus on ncov2019 (Github; https://github.com/connor-lab/ncov2019-artic-nf).

28. D. J. Baker, A. Aydin, T. Le-Viet, G. L. Kay, S. Rudder, L. de Oliveira Martins, A. P. Tedim, A. Kolyva, M. Diaz, N.-F. Alikhan, L. Meadows, A. Bell, A. V. Gutierrez, A. J. Trotter, N. M. Thomson, R. Gilroy, L. Griffith, E. M. Adriaenssens, R. Stanley, I. G. Charles, N. Elumogo, J. Wain, R. Prakash, E. Meader, A. E. Mather, M. A. Webber, S. Dervisevic, A. J. Page, J. O'Grady, CoronaHiT: High-throughput sequencing of SARS-CoV-2 genomes. *Genome Med.* **13**, 21 (2021). doi:10.1186/s13073-021-00839-5

29. "pangolin: Software package for assigning SARS-CoV-2 genome sequences to global lineages" (Github; https://github.com/cov-lineages/pangolin).

30. M. D. Hoffman, A. Gelman, The no-U-Turn Sampler: Adaptively setting path lengths in Hamiltonian Monte Carlo. *J. Mach. Learn. Res.* **15**, 1593–1623 (2014).

31. S. Lang, A. Brezger, Bayesian P-Splines. *J. Comput. Graph. Stat.* **13**, 183–212 (2004). doi:10.1198/1061860043010
